# Supplementary figures and images for: Prognostic model for survival in patients with neuroendocrine carcinomas of the cervix: SEER database analysis and a single-center retrospective study
Source: PLoS One. 2024 Jan 5;19(1):e0296446. doi: 10.1371/journal.pone.0296446 (PMC10769015; doi:10.1371/journal.pone.0296446)

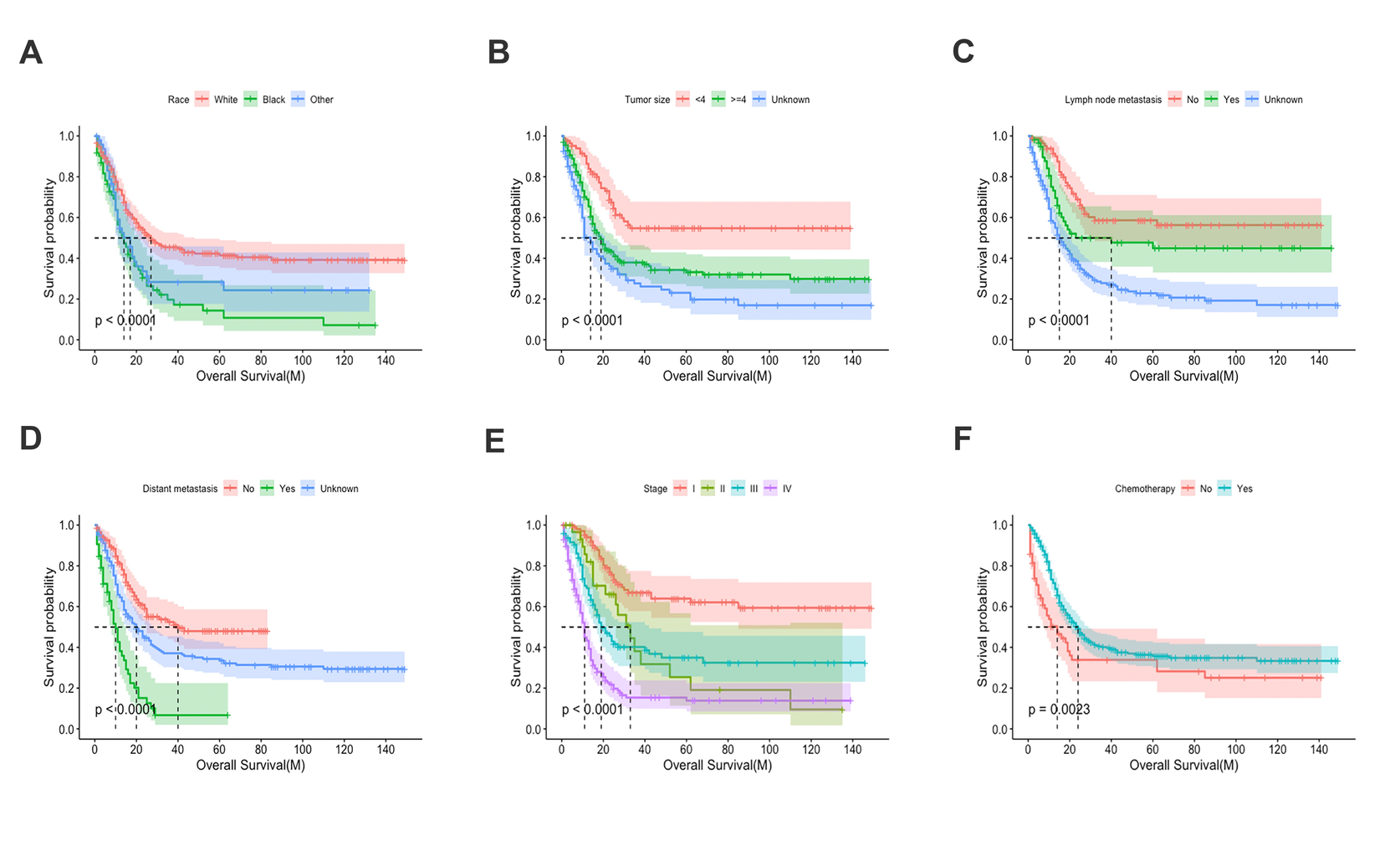

Supplement: S1 Fig — A, Race; B, Tumor size; C, Lymph node metastasis; D, Distant metastasis; E, Stage; F, Chemotherapy. (TIF) [file pone.0296446.s001.tif]

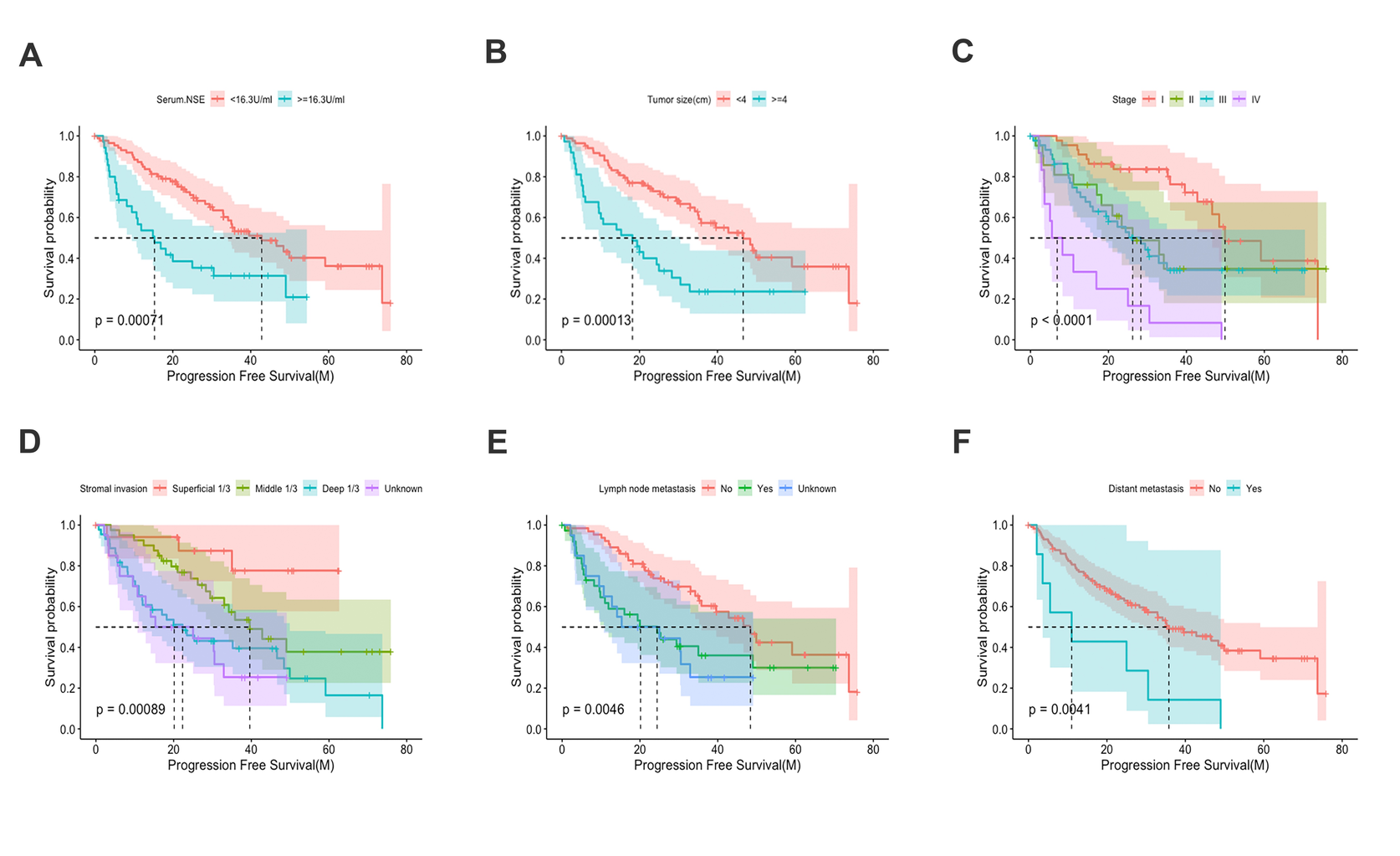

Supplement: S2 Fig — A, Serum NSE; B, Tumor size; C, Stage; D, Stromal Invasion; E, Lymph node metastasis; F, Distant metastasis. (TIF) [file pone.0296446.s002.tif]

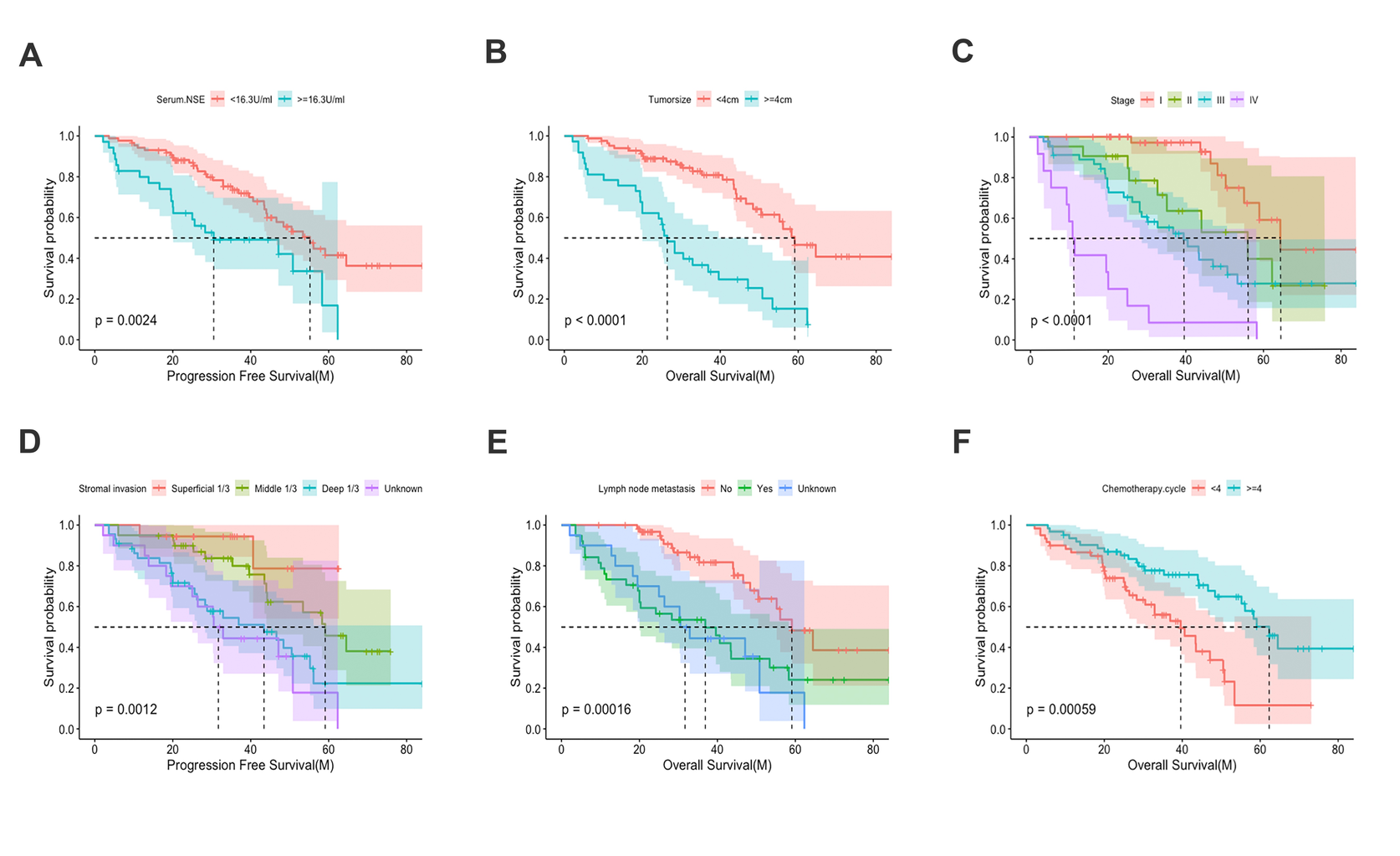

Supplement: S3 Fig — A, Serum NSE; B. Tumor size; C, Stage; D, Stromal Invasion; E, Lymph node metastasis; F, Cycles of chemotherapy. (TIF) [file pone.0296446.s003.tif]
