# Supplementary material for: Prognostic model for survival in patients with neuroendocrine carcinomas of the cervix: SEER database analysis and a single-center retrospective study
Source: PLoS One. 2024 Jan 5;19(1):e0296446. doi: 10.1371/journal.pone.0296446 (PMC10769015; doi:10.1371/journal.pone.0296446)
Supplement: S1 Table — (DOCX) [file pone.0296446.s004.docx]

**Table S1** The clinicopathologic characteristics of NECC patients in the external validation cohort

| **Variables** |  | Total (N=122) | |
| --- | --- | --- | --- |
|  |  | n | % |
| Age | <44 | 56 | 45.9 |
|  | ≥44 | 66 | 54.1 |
| Menopausal status | No | 80 | 65.6 |
|  | Yes | 42 | 34.4 |
| Serum NSE level | ＜16.3 | 87 | 71.3 |
|  | ≥16.3 | 35 | 38.7 |
| Histology | Pure | 109 | 89.3 |
|  | Mixed | 13 | 10.7 |
| Tumor size (cm) | ＜4 | 85 | 69.7 |
|  | ≥4 | 37 | 30.3 |
| Stromal Invasion | Superficial 1/3 | 18 | 14.7 |
|  | Middle 1/3 | 40 | 32.7 |
|  | Deep 1/3 | 44 | 36.1 |
|  | Unknown | 20 | 16.4 |
| LVSI | No | 79 | 64.8 |
|  | Yes | 23 | 18.9 |
|  | Unknown | 20 | 16.4 |
| Lymph node metastasis | No | 64 | 52.5 |
|  | Yes | 38 | 31.1 |
|  | Unknown | 20 | 16.4 |
| Distant metastasis | No | 115 | 94.2 |
|  | Yes | 7 | 5.8 |
| Stage (FIGO 2018) | Ⅰ | 44 | 36.1 |
|  | Ⅱ | 21 | 17.2 |
|  | Ⅲ | 45 | 36.9 |
|  | Ⅳ | 12 | 9.8 |
| Neoadjuvant chemotherapy | No | 79 | 64.8 |
|  | Yes | 23 | 18.9 |
| Chemotherapy  (Primary/Adjuvant only) | No | 17 | 13.9 |
|  | Yes | 105 | 86.1 |
| Chemotherapy cycles Primary/Adjuvant only) | ＜4 | 60 | 49.2 |
|  | ≥4 | 62 | 50.8 |
| Radiation | No | 47 | 38.5 |
|  | Yes | 75 | 61.5 |
| Syn | Negative | 10 | 8.2 |
|  | Positive | 112 | 91.8 |
| CgA | Negative | 40 | 32.8 |
|  | Positive | 76 | 62.2 |
|  | Not examined | 6 | 5.0 |
| NSE | Negative | 13 | 10.7 |
|  | Positive | 34 | 27.8 |
|  | Not examined | 75 | 61.5 |
| CD56 | Negative | 25 | 20.5 |
|  | Positive | 55 | 45.1 |
|  | Not examined | 42 | 34.4 |
| Ki-67 | <60% | 44 | 36.1 |
|  | ≥60% | 21 | 17.2 |
|  | Not examined | 57 | 46.7 |
