# Supplementary material for: Prognostic model for survival in patients with neuroendocrine carcinomas of the cervix: SEER database analysis and a single-center retrospective study
Source: PLoS One. 2024 Jan 5;19(1):e0296446. doi: 10.1371/journal.pone.0296446 (PMC10769015; doi:10.1371/journal.pone.0296446)
Supplement: S2 Table — * P-value <0.05. (DOCX) [file pone.0296446.s005.docx]

**Table S2** Univariate analysis of immunohistochemical markers of 3-year progression-free survival and 3-year overall survival of NECC patients in the external validation cohort (N=122)

| **Variables** |  | **3-year PFS %** | **χ^2^** | ***p* value** | **3-year OS %** | **χ^2^** | ***p* value** |
| --- | --- | --- | --- | --- | --- | --- | --- |
| Syn | Negative | 0.0 | 10.613 | 0.001^*^ | 66.9 | 1.233 | 0.267 |
|  | Positive | 50.7 |  |  | 18.0 |  |  |
| CgA | Negative | 44.9 | 0.055 | 0.814 | 68.5 | 0.991 | 0.320 |
|  | Positive | 51.4 |  |  | 63.9 |  |  |
| NSE | Negative | 44.9 | 0.004 | 0.948 | 71.7 | 0.001 | 0.977 |
|  | Positive | 42.0 |  |  | 52.7 |  |  |
| CD56 | Negative | 33.1 | 0.551 | 0.458 | 60.6 | 0.561 | 0.454 |
|  | Positive | 48.5 |  |  | 67.0 |  |  |
| Ki-67 | Negative | 54.9 |  |  | 69.3 | 0.420 | 0.517 |
|  | Positive | 53.8 | 0.148 | 0.700 | 65.6 |  |  |

**p*<0.05
