# Supplementary material for: Prognostic model for survival in patients with neuroendocrine carcinomas of the cervix: SEER database analysis and a single-center retrospective study
Source: PLoS One. 2024 Jan 5;19(1):e0296446. doi: 10.1371/journal.pone.0296446 (PMC10769015; doi:10.1371/journal.pone.0296446)
Supplement: Attachment — Submitted filename: Response to reviewers.docx [file pone.0296446.s006.docx]

Rebuttal letter

Dear Editor Andrea Giannini,

Thank you for all your effort made on our manuscript entitled “A Prognostic model for survival in patients with neuroendocrine carcinomas of the cervix: SEER database analysis and a single-center retrospective study.” All authors listed have been carefully discussing the comments we received from the reviewers and making revision accordingly in our revised manuscript, and you will find our responses to each comment on the next page. We also thoroughly revised our manuscript and cover letter according to *PLOS ONE*'s style requirements, and hopefully all the typos and grammar errors in the former manuscript have also been corrected accordingly. We really appreciate the contribution of all reviewers and hope this revised manuscript is acceptable for publication on *PLOS ONE*.

If there is any clarification or further requirements needed on this revised version, please kindly let us know.

Sincerely Yours,

Zheng Li

The Third Affiliated Hospital of Kunming Medical University

E-mail: lizheng@kmmu.edu.cn

October 25th, 2023

**Reviewer: 1**

In this manuscript, the authors successfully established and validated a prognostic model based on SEER data base for patients with NECC. The retrospective study for patients with NECC also be processed. Retrospective studies have also well validated this prognostic model.

**Response：**We appreciate the reviewer’s positive comments on our manuscript.

**Reviewer: 2**

1) In line 191 to 192, you mentioned the cumulative 3-year progression-free rate was ‘3.2 %’, which was fewer than the 5-year rate, 35.6 %. Please check the number.

**Response**: We appreciate the reviewer’s carefully checking. We checked the raw data and found out the cumulative progression-free survival rate of 3 years at YCH should be 53.2%. We believe it was a typo and have corrected it in the revised manuscript.

2) In line 275, you should correct the word ‘fund’ to ‘found’.

**Response**: We have made modifications in the corresponding positions in the revised manuscript as the reviewer suggested. Thanks for pointing out another typo.

3) In line 254 and 256, SNCEC should be correct to SCNEC.

**Response**: Yes, it should be “SCNECC” instead of “SNCEC,” and we have made the correction accordingly for all these acronyms in the revised manuscript.

4) In Table 3, 4, there are ‘chemotherapy’ and ‘Chemotherapy’ in the Variables, which make the readers confused. If possible, it may be better to add some annotations.

**Response**: We use “Chemotherapy” in Table 3 and 4 in the Variables lines to make it more clearly in the revised manuscript.

5) Not only internal validation cohort using SEER data, but also external validation cohort using YCH data, there are no merits to treat using radiotherapy for the patients suffering from NECC cervical canecr. In general radiotherapy is useful and helps to improve the prognosis for the patients suffering from cervical cancer, however, it is different for the NECC cervical cancer. Although you mentioned these comments in discussion, you should announce the fact using strong words.

**Response**: Yes, and our latest multi-centers retrospective study published on Lancet Oncology confirmed that even for patients with local advanced stage of NECC, they are more likely benefit from surgery rather than radiation, and we added a short graph in discussion part to emphasize that idea with a new reference [30]. (line in revised manuscript).

**Reviewer: 3**

I read with great interest the manuscript, which falls within the aim of this Journal and offers a high-quality overview of the topic.
Although the manuscript can be considered already of high quality, I would suggest to take into account the following minor recommendations:
- I suggest another round of language revision, in order to correct few typos and improve readability.

**Response**: We believe we have corrected all the typos and gramma errors, and we rewrote the introduction, as well as material and method section in the revised manuscript to improve its readability.

- I find it interesting to include a reference to the screening programs for early cervical cancer diagnosis (Golia D'Augè T, Giannini A, Bogani G, Di Dio C, Laganà AS, Di Donato V, Salerno MG, Caserta D, Chiantera V, Vizza E, Muzii L, D’Oria O. Prevention, Screening, Treatment and Follow-Up of Gynecological Cancers: State of Art and Future Perspectives. Clin. Exp. Obstet. Gynecol. 2023, 50(8), 160. <https://doi.org/10.31083/j.ceog5008160>).
**Response**: We really appreciate that idea and believe that is a decent paper. But after careful consideration, we finally decided not to add this review as a new reference since this manuscript focused only on the establishment and confirmation of prognostic model for survival in patients with NECC, and we will cite this review on our other papers concerning the screening programs of cervical cancer.

- The authors have not adequately highlighted the strengths of their study. I suggest better specifying these points.

**Response**: We highlighted the strengths of our study in the revised introduction and discussion sections in the revised manuscript, thanks for the remind.

**Reviewer: 4**

1） ON what basis was a 7:3 (training: validation) selection made?

**Response**: As a matter of fact, there is no consensus on the optimal partition ratio of training set versus validation set in the establishment of prediction model till now. In previous studies, the partition ratio of the training set and validation set ranges from 5:5 to 7:3. However, the more accurate the model is, the larger sample size it requires. Taken that the accuracy of the model we established is about 98%, a ratio of 5:5 will require at least 3,000 patients in each set, and that will not be practical in the current study. That is why we finally chose a partition ratio of 7:3 to assign more participants in the training set to improve the model’s accuracy in a relatively small sample size, and we added two more extra reference to demonstrate how that choice are made (reference 19, 20).

2） Cox proportional hazards model (and coxph() function) was used for nomogram constructions; how was the proportionality assumption assessed?

**Response**: In the current study, five clinicopathologic factors (race, tumor size, distant metastasis, stage, and chemotherapy) were selected by multivariate analyses based on Cox proportional hazards model and then were enrolled in nomogram construction. As far as we know, all the clinicopathologic factors mentioned above (except race）are graded variables and Cox proportional hazards model was the most common and reasonable tool to analyze the impact those graded variables on survival by a dose-dependent manner (for example, tumor size, HR=1.445; distant metastasis, HR=1.804; chemotherapy, HR=0.513), as most previous studies have been done (reference 5-9). Then the nomogram (especially the four factors one excluding race) we constructed is also based on the proportionality assumption, because the variables that built the nomogram are all graded variables, so they exert their impact on survival on a dose-dependent manner as we mentioned before, and we believe that is why other previous studies made the same proportionality assumption when they built their nomograms in their works (reference 17, 18).

3） The variable selection carried out in Results (Subsection 2) appeared somewhat adhoc; any reason, why any formal method (like Lasso, etc) were not used?

**Response**: Thanks for your kindly and professional advice concerning statistic processes. We believe that the selection of variable in medical studies like the current one, is based on both scientific reasons and statistical ones. In this particular case, as we mentioned in “Univariate and multivariate analysis in the training cohort” (Results, subsection 2), age, race, tumor size, distant metastasis, stage, LNM and chemotherapy were variables that were selected through univariate analysis, but we decided not to put LNM in the multivariate analysis because the variable “stage” (AJCC stage but not FIGO stage in the training set) here, is actually containing the information of LNM, and that explained why a strong collinear relationship between LNM and stage were observed. Furthermore, there were 62.5% of patients in training cohort (235/376) whose LNM statuses were marked as “unknown”, and any statistical process based on such a high deletion rate is risky. So, we decided not to enroll “LNM” in the multivariate analysis but keep “stage” in since the latter contains more information rather than the former. Furthermore, we usually employed Lasso in multivariate analysis when there were too many variables (usually 10 or more) stood from univariate analysis and we kept deleting variables for scientific reasons. We also rewrote Results, subsection 2 in the revised manuscript to explain our selection of variables more clearly, hopefully that will not cause any ad hoc feelings for our reviewers and readers anymore.
