# Supplementary material for: Prognostic model for survival in patients with neuroendocrine carcinomas of the cervix: SEER database analysis and a single-center retrospective study
Source: PLoS One. 2024 Jan 5;19(1):e0296446. doi: 10.1371/journal.pone.0296446 (PMC10769015; doi:10.1371/journal.pone.0296446)
Supplement: Attachment — Submitted filename: Response to reviewers.docx [file pone.0296446.s007.docx]

Rebuttal Letter

Dear Editor Andrea Giannini,

Thank you for your extra efforts made on our manuscript entitled “A Prognostic model for survival in patients with neuroendocrine carcinomas of the cervix: SEER database analysis and a single-center retrospective study.” In the current revised manuscript, we consulted our expert on statistics and addressed the problem Reviewer 4 proposed. We really appreciate the contribution of all reviewers and hope this revised manuscript is acceptable for publication on *PLOS ONE*.

If there is any clarification or further requirements needed on this revised version, please kindly let us know.

Sincerely Yours,

Zheng Li

The Third Affiliated Hospital of Kunming Medical University

E-mail: lizheng@kmmu.edu.cn

November 26th, 2023

**Reviewer #4:** The authors addressed my previous comments, with a great degree of satisfaction. However, the reply on "checking proportional hazards assumptions" was not adequate. Notethat, there are formal tests to assess the assumption; see here: https://stat.ethz.ch/R-manual/R-devel/library/survival/html/cox.zph.html

I understand that failure of those assumptions may lead to exploring a different model, suich as the accelerated failure time, etc, which may not have the elegant PH interpretations (in terms of hazard ratios). To provide some statistical rigor to this manuscript, the authors are advised either to formally check the assumptions. Even if the assumptions fail, they may comment that for the sake of interpretability, they are sticking to the Cox PH model, but future research may pertain to exploring alternative modeling

**Response**: Thank you for your feedback and for providing the link to the formal tests for checking proportional hazards (PH) assumptions. We apologize for not adequately addressing this concern in our previous response, and we understand the importance of assessing the assumptions and the potential implications for model interpretation. Considering your suggestion, we consulted our expert on statistics (Pro. Min Zhao) and employed two methods (Global test and Schoenfeld residual graph) to check the assumptions. Firstly, we run the Cox.zph function in the R language survival package for Global test. If the P-values of each covariate are greater than 0.05, it indicates that each variable meets the PH test, and while the overall test P-value of the model is also greater than 0.05, it suggests the model meets the PH test.

Here are our output results:

chisq df P-value

Race	0.0829 1 0.773

Stage	3.1172 1 0.077

Tumorsize	2.8043 1 0.094

Distant.metastasis	0.5762 1 0.448

Chemotherapy	0.1177 1 0.732

GLOBAL	6.2295 5 0.285

Now we can find out that each covariate, as well as the global test, is of P-value greater than 0.05, which means that the Cox model conforms to PH assumption.

Then we used the ggcoxzph() function to generate a normalized Schoenfeld residual correlation image concerning time for each covariate, and the output image is as follows：

In the above figures, solid lines represent the smooth spline fitting of the curve, while the dashed line represents the+/-2 times standard error range around the fitting. Because we assume that the coefficient β 1, β 2, and β 3 does not change over time, from the graph inspection, the covariates we included vary over time, which means all covariates satisfy the assumption of PH.

To sum up, we employed two formal tests as the reviewer indicated to check whether our model met the proportional hazards (PH) assumptions and it turns out the model we built did meet the assumption. So, we added a short sentence in the “Statistical methods” part in the current revised manuscript (lines 132 to 134) to indicate that idea.

Thank you again for bringing this to our attention, and we will ensure that the manuscript includes a more thorough discussion on checking proportional hazards assumptions and the potential implications for future research.
